# Radiomic Features From Diffusion-Weighted MRI of Retroperitoneal Soft-Tissue Sarcomas Are Repeatable and Exhibit Change After Radiotherapy

**DOI:** 10.3389/fonc.2022.899180

**Published:** 2022-07-18

**Authors:** Imogen Thrussell, Jessica M. Winfield, Matthew R. Orton, Aisha B. Miah, Shane H. Zaidi, Amani Arthur, Khin Thway, Dirk C. Strauss, David J. Collins, Dow-Mu Koh, Uwe Oelfke, Paul H. Huang, James P. B. O’Connor, Christina Messiou, Matthew D. Blackledge

**Affiliations:** ^1^ Division of Radiotherapy and Imaging, The Institute of Cancer Research, London, United Kingdom; ^2^ Department of Radiology, The Royal Marsden National Health Service (NHS) Foundation Trust, Sutton, United Kingdom; ^3^ Sarcoma Unit, The Royal Marsden National Health Service (NHS) Foundation Trust, London, United Kingdom; ^4^ Department of Histopathology, The Royal Marsden National Health Service (NHS) Foundation Trust, London, United Kingdom; ^5^ Department of Surgery, The Royal Marsden National Health Service (NHS) Foundation Trust, London, United Kingdom; ^6^ Division of Molecular Pathology, The Institute of Cancer Research, London, United Kingdom; ^7^ Division of Cancer Sciences, University of Manchester, Manchester, United Kingdom; ^8^ Department of Radiology, The Christie Hospital, Manchester, United Kingdom

**Keywords:** radiomics, soft-tissue sarcoma, radiotherapy, DWI (diffusion weighted imaging), Intraclass correlation coefficient (ICC), repeatability, apparent diffusion coefficient (ADC)

## Abstract

**Background:**

Size-based assessments are inaccurate indicators of tumor response in soft-tissue sarcoma (STS), motivating the requirement for new response imaging biomarkers for this rare and heterogeneous disease. In this study, we assess the test–retest repeatability of radiomic features from MR diffusion-weighted imaging (DWI) and derived maps of apparent diffusion coefficient (ADC) in retroperitoneal STS and compare baseline repeatability with changes in radiomic features following radiotherapy (RT).

**Materials and Methods:**

Thirty patients with retroperitoneal STS received an MR examination prior to treatment, of whom 23/30 were investigated in our repeatability analysis having received repeat baseline examinations and 14/30 patients were investigated in our post-treatment analysis having received an MR examination after completing pre-operative RT. One hundred and seven radiomic features were extracted from the full manually delineated tumor region using PyRadiomics. Test–retest repeatability was assessed using an intraclass correlation coefficient (baseline ICC), and post-radiotherapy variance analysis (post-RT-IMS) was used to compare the change in radiomic feature value to baseline repeatability.

**Results:**

For the ADC maps and DWI images, 101 and 102 features demonstrated good baseline repeatability (baseline ICC > 0.85), respectively. Forty-three and 2 features demonstrated both good baseline repeatability and a high post-RT-IMS (>0.85), respectively. Pearson correlation between the baseline ICC and post-RT-IMS was weak (0.432 and 0.133, respectively).

**Conclusions:**

The ADC-based radiomic analysis shows better test–retest repeatability compared with features derived from DWI images in STS, and some of these features are sensitive to post-treatment change. However, good repeatability at baseline does not imply sensitivity to post-treatment change.

## Introduction

Soft-tissue sarcomas (STS) are rare tumors of the connective tissues and account for 1% of all cancers (1). While the radiological assessment of STS typically includes size-based criteria, such as those defined by the Response Evaluation Criteria in Solid Tumors guidelines (RECIST 1.1) (2), intralesion heterogeneity is commonly seen in the clinic, both in tumor appearance and treatment response (3). Furthermore, several studies have reported that changes in tumor size have a poor correlation with histopathological tumor response (4–8). This has led to guidelines being published by The European Organisation for Research and Treatment of Cancer (EORTC) Soft Tissue and Bone Sarcoma Group (9), where it is recommended that size and volume measurements should not be used to reflect histopathological response following treatment (except for myxoid liposarcomas). There is therefore an urgent need to develop robust clinical imaging biomarkers (IBs) that i) better reflect histopathological change and ii) capture intralesion heterogeneity before, during, and after treatment.

Quantitative diffusion-weighted imaging (DWI) is showing increased utility for monitoring response in STS (10). Measurements of the apparent diffusion coefficient (ADC) calculated from DWI have demonstrated an inverse correlation with tissue cellularity (11) and thus could act as a surrogate IB for the early assessment of radiotherapy treatment response (12). A key advantage of such quantitative techniques includes the fact that derived maps are representative of tumor biology and thus may offer deeper insights into the heterogeneous patterns of tumor response. In a previous cohort study of patients with retroperitoneal STS, median ADC after radiotherapy demonstrated a significant increase compared to baseline and 4/14 patients showed an increase in median ADC outside 95% repeatability limits of agreement (10). However, the assessment of total tumor ADC failed to capture the spatial heterogeneity within these lesions, obscuring the interpretation of changes following treatment (13).

Radiomic analysis extracts a set of mathematical features describing the relationships and patterns between pixels that quantify image characteristics such as texture, intensity, and shape. Radiomic features are thought to reflect the heterogeneity of underlying biological features within the tumor such as necrosis, vascularity, and histological variation (14, 15). In a recent study investigating ADC-based radiomic features in STS by Corino et al., differences were observed in radiomic feature values between intermediate- and high-grade lesions (16). Lee et al. observed that ADC-based radiomic features may quantify tumor heterogeneity, although they did not find an improvement in diagnosing benign and malignant STS compared to ADC alone (17).

Recently, changes between pre- and post-treatment radiomic features (delta-radiomics) have been associated with tumor response; Gao et al. demonstrated that ADC-based delta-radiomics improved response prediction in STS following pre-operative radiotherapy treatment using a support vector machine (SVM) model (18). While these data are encouraging, they are limited without evaluation of the repeatability of ADC-based radiomic features, as outlined in recent consensus recommendations for the clinical translation of response IBs (19). To the best of our knowledge, there exists no study assessing the test–retest repeatability of ADC-based radiomics in STS.

This study has two main aims. Firstly, we aim to assess the test–retest stability of radiomic features derived from ADC measurements (quantitative imaging) and compare this with the corresponding features derived from low *b*-value DW images (qualitative imaging) in a cohort of patients with retroperitoneal STS. Secondly, as baseline repeatability is not necessarily related to sensitivity to response (20), we introduce a novel metric that compares the baseline repeatability of radiomic features with their ability to demonstrate change following treatment. We then use this methodology to identify a set of radiomic features that are both highly repeatable and sensitive to post-treatment change for use in prospective clinical STS studies.

## Materials and Methods

This study was reviewed and approved by the Royal Marsden Hospital committee for clinical research and approval from a national Research Ethics Committee (East of England—Cambridge East Research Ethics Committee).

### Patient Population, Imaging, and Radiotherapy Schedule

**Figure 1:**
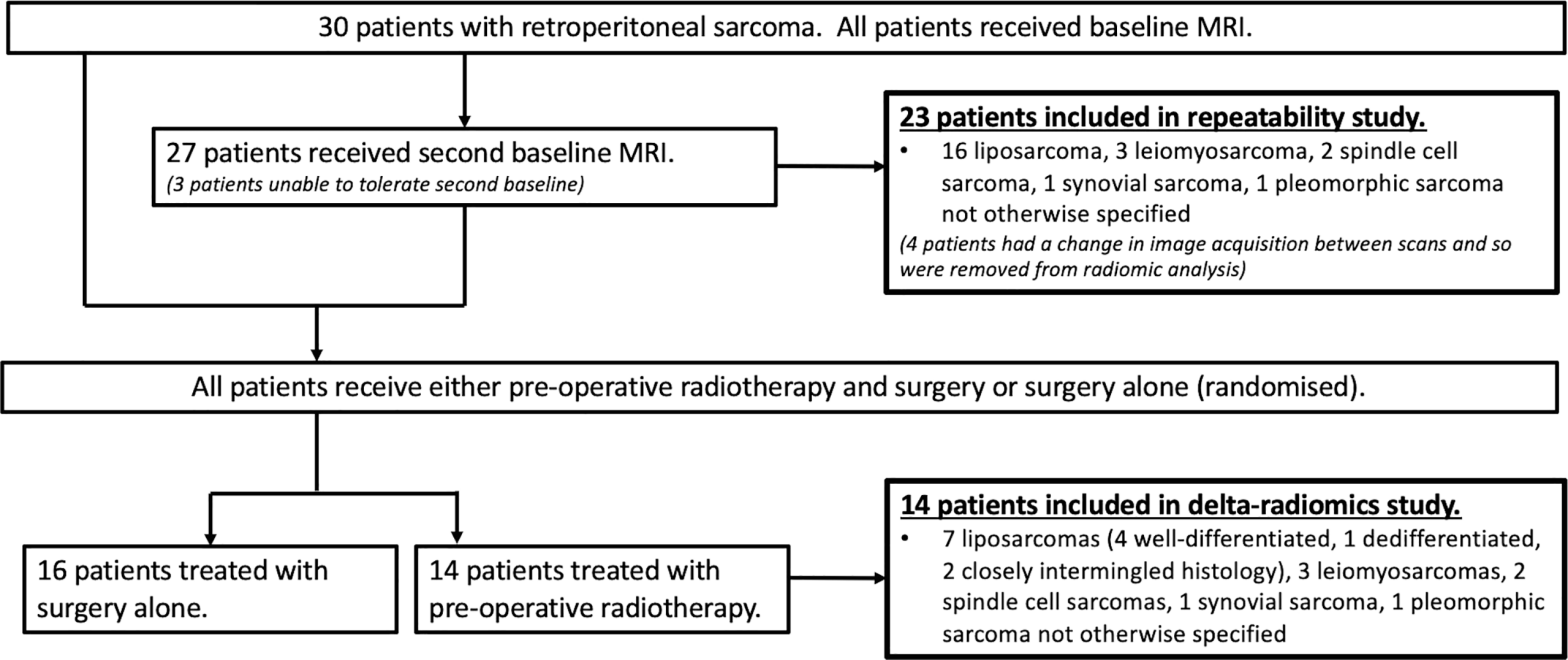
Flowchart of the study population. Flowchart showing the numbers of patients and histology included in each of the repeatability and delta-radiomics analysis sections of the study. Figure adapted from (10).

Thirty patients with retroperitoneal STS received MR examinations on a 1.5-T MR scanner before treatment (MAGNETOM Aera, Siemens Healthcare, Erlangen, Germany). Imaging included axial DWI with *b*-values of 50, 600, and 900 s/mm^2^. Full details of the study, patient protocol, and imaging protocol have been reported previously by Winfield et al. (10). Twenty-seven of 30 patients were repositioned and then received a repeat baseline DWI acquisition in the same scan session. Four of these patients were excluded from the repeatability analysis due to a change in image acquisition parameters during the second baseline acquisition [for two patients, the imaging field of view (FoV) was reduced for the second scan for patient comfort, and two patients had a change in image intensity between repeat scans]. Fourteen of 30 patients were treated with radiotherapy; these patients received at least one baseline MR scan and another after completing radiotherapy treatment, prior to surgery. **Figure 1** shows the study organization of patients included in each of the repeatability and delta-radiomics sections of this analysis. **Supplementary Material A** presents the breakdown of STS subtypes studied and whether they received a second baseline and/or post-radiotherapy scan. For those treated with radiotherapy, 28 daily fractions were administered over 5.5 weeks delivering a median dose of 50.4 Gy.

### Image Processing and Radiomic Feature Extraction

Regions-of-interest (ROIs) were delineated on every slice in which the tumor appeared on axial T_2_-weighted images, using inhouse software by experienced soft-tissue sarcoma radiologist (CM) with over 10 years of experience and transferred to all imaging series.

ADC maps were created using a least-squares monoexponential fit (21). ROIs were transferred onto the calculated ADC maps and subsequently converted into binary mask segmentations. To allow for direct comparison between quantitative and non-quantitative imaging, the ROIs were also transferred onto the *b* = 50 s/mm^2^ diffusion-weighted images (hereafter referred to as b50); b50 was chosen as it had the highest signal-to-noise ratio compared with the *b* = 600 and 900 s/mm^2^ images. As some patients required different imaging FoVs to the standard protocol, images and segmentations were resampled to have matching voxel sizes (2.375, 2.375, 5.0 mm) across all patients (ADC and b50 images were resampled using linear interpolation, and masks were resampled using nearest neighbor interpolation) and stacked to create 3D volumes (22, 23). To generate additional image sets, histogram equalization, which spreads out pixel intensity levels resulting in heightened image contrast and texture, was applied to the ADC maps and b50 images within the delineated tumor regions. The histogram-equalized images were scaled by 300 to match the gray level range of the ADC images [units of 10^−5^ mm^2^ s^−1^ were used to match previously published work (24)]. The original b50 images were not rescaled as the gray level range was already within the same order of magnitude. Radiomic feature extraction was performed on the four different sets of images: i) ADC maps, ii) b50 images, iii) histogram-equalized ADC maps, and iv) histogram-equalized b50 images. The open-source package PyRadiomics (25) (v3.0.1.) was used to extract radiomic features from all four image contrasts in 3D: 18 first-order, 75 second-order (glcm, gldm, glrlm, glszm, ngtdm), and 14 shape features. The following settings were used across all image contrasts: bin width = 10 and force2D = True (due to the anisotropic voxel dimensions). No wavelet or other filtering operations were performed.

### Calculation/Theory

We denote *x_ikl_* as the *i*-th radiomic feature, for the *k*-th patient and *l*-th baseline measurement. From the natural logarithm of these values, yikl = ln(xikl), the following repeatability statistics were derived.

The baseline within-subject standard deviation for *N* patients is defined as ${\text{s}}_{{\text{w}}_{\text{bs}}}=\sqrt{\frac{1}{2N}\sum_{k=1}^{N}{\left(y_{ik2}-y_{ik1}\right)}^{2}}$ . The between-subject mean squares (BMS) and within-subject mean squares (WMS) are calculated, respectively, as $\text{BMS}=\frac{2}{N}\;{\sum_{k=1}^{N}\left({\bar{y}}_{ik}-{\bar{\bar{y}}}_{i}\right)}^{2}$ and $\text{WMS}=\frac{1}{N}\;\sum_{k=1}^{N}\left[{\left(y_{ik1}-{\bar{y}}_{ik}\right)}^{2}+{\left(y_{ik2}-{\bar{y}}_{ik}\right)}^{2}\right]$ , where ${\bar{y}}_{ik}=\frac{y_{ik1}+y_{ik2}}{2}$ and ${\bar{\bar{y}}}_{ik}=\frac{1}{N}\sum_{k=1}^{N}{\bar{y}}_{ik}$ . The baseline between-subject standard deviation is defined as $s_{{\text{b}}_{\text{bs}}}=\sqrt{\frac{\text{BMS}-\text{WMS}}{2}}$ , from which the baseline intraclass correlation coefficient (ICC) is derived as baseline- $\text{ICC}=\frac{s_{{\text{b}}_{\text{bs}}}^{2}}{s_{{\text{b}}_{\text{bs}}}^{2}+s_{{\text{w}}_{\text{bs}}}^{2}}$ . Bland–Altman plots were derived for the features, with 95% limits of agreement (LoA) plotted on absolute scales (26, 27) where: $\text{LoA}=\left[\text{exp}\left(-1.96\;s_{{\text{w}}_{\text{bs}}}\sqrt{2}\right)-1,\text{exp}\left(+1.96\;s_{{\text{w}}_{\text{bs}}}\sqrt{2}\right)-1\right]$ . Two of the radiomic features (Skewness and glcmClusterShade) returned both positive and negative values and thus could not be analyzed using the natural logarithm of their values. For these two features, the analysis was performed on the raw data and the LoA was calculated as follows: $\text{LoA}=\pm 1.96\;\times \sqrt{\frac{1}{N}\sum_{k=1}^{N}{\left(x_{ik2}-x_{ik1}\right)}^{2}}$ . Defining *y_ik_*_3_ as the natural logarithm of the *i*-th radiomic feature calculated after radiotherapy and *M* as the number of patients that had both baseline and post-RT MR examinations, the within-subject standard deviation after radiotherapy was calculated as $s_{{\text{w}}_{\text{rt}}}=\sqrt{\frac{1}{2M}\sum_{k=1}^{M}{\left(y_{ik3}-y_{ik1}\right)}^{2}}$ . A post-radiotherapy variance analysis metric [named as intermeasurement sensitivity (IMS)] was then calculated as $\text{postRT}-\text{IMS}=\frac{s_{{\text{w}}_{\text{rt}}}^{2}}{s_{{\text{w}}_{\text{rt}}}^{2}+s_{{\text{w}}_{\text{bs}}}^{2}}$ (for Skewness and glcmClusterShade where log values could not be used, the above analysis was performed on the raw data). A post-RT-IMS metric close to 1 occurs when the variance between feature value before and after radiotherapy is much greater than the variance between baseline scans, indicating that these features are repeatable within the context of changes expected after treatment. A graphical representation of the metrics derived is illustrated in **Supplementary Material B**.

### Statistical Analysis

For all image contrasts, the number of features that satisfied two different criteria was identified:

(i) Good repeatability: Radiomic feature had a baseline ICC greater than 0.85 (28).

(ii) Substantial change after treatment: Radiomic feature had a post-RT-IMS greater than 0.85.

To determine whether the baseline ICC is indicative of the post-RT-IMS, the Pearson correlation coefficient between both measurements across all features (PCC) was calculated for each image contrast.

### Post-RT Fractional Changes

To quantify post-RT changes in radiomic feature *i* for patient *k*, we define the fractional change as ${\text{Δ}}_{ik}=\frac{x_{ik3}-x_{ik1}}{x_{ik1}}$ .

### Independent Subset

The values of the radiomic features from the first baseline scan were used to form a matrix of Pearson product-moment correlation coefficients, $\;r_{ij}=\frac{C_{ij}}{\sqrt{C_{ii}*C_{jj}}}$ , where *C_ij_* is the covariance between features *i* and *j*. Higher agglomerative clustering was performed to obtain cluster groups of strongly correlated features (hereafter referred to as correlation groups) using seaborn (v0.9.0) and scipy (v1.3.1) (29–31) with the following settings: $\text{distance metric }=\;1-r_{ij}^{2}$ , cluster method = average, and cluster distance cutoff = 0.5. We assume features from different correlation groups to be independent. The correlation groups for the non-histogram-equalized ADC-based radiomic features are in **Supplementary Material C.**

To explore a small subset of the features in more detail, a subset of independent features that demonstrated change post-treatment (the independent delta-radiomics subset) was identified by selecting the feature, within each correlation group, with the highest post-RT-IMS that satisfied criteria (i) and (ii).

## Results

**Figure 2:**
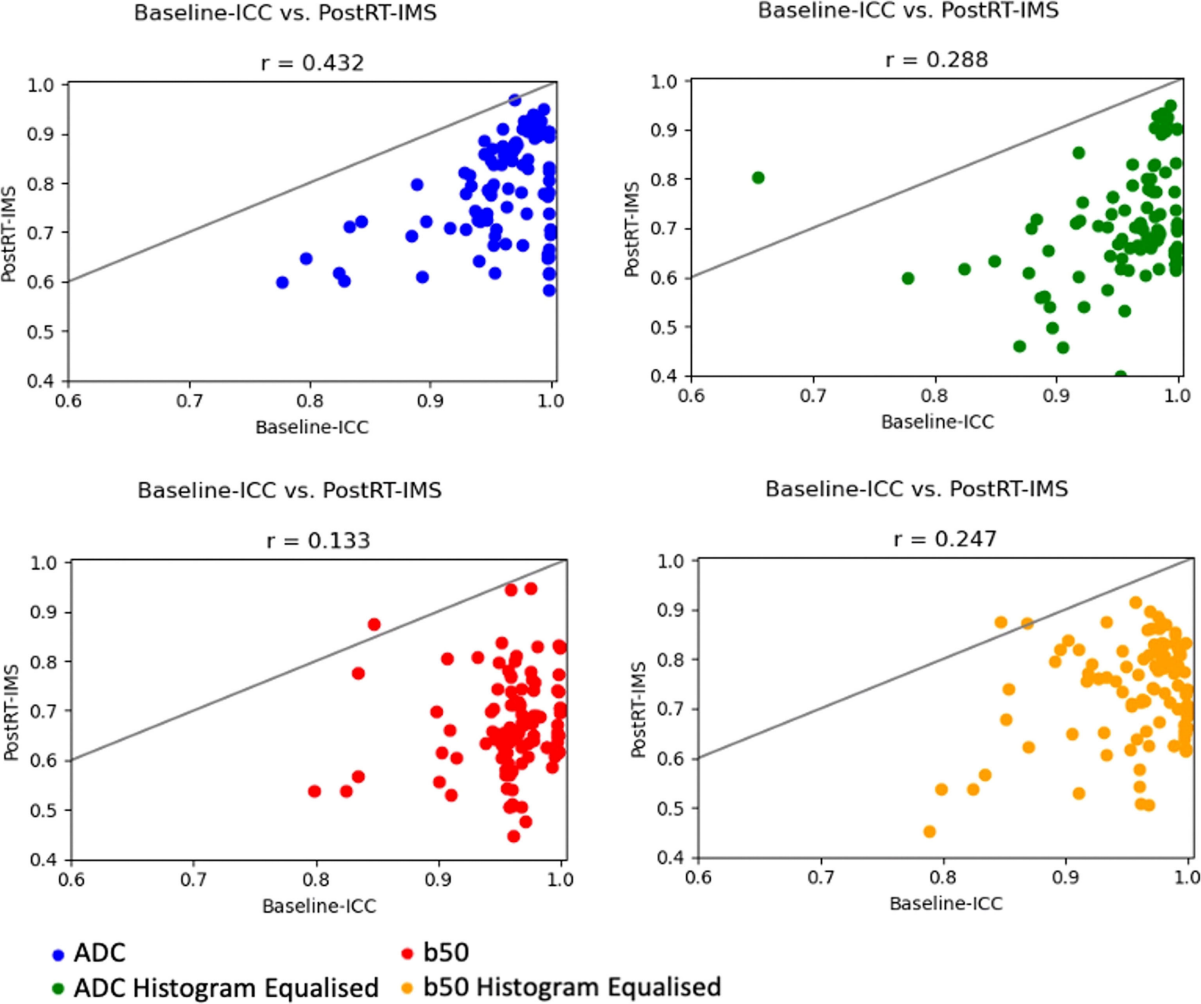
ICC–IMS correlation plots. Correlation plots showing the baseline ICC vs. the post-RT-IMS for all radiomic features for each image type. A solid line of equality is shown in each. The Pearson correlation coefficient (*r*) is shown as a subheading.

For non-histogram-equalized ADC maps, 101/107 radiomic features demonstrated high baseline ICC [criterion (i)], of which 43 features demonstrated high post-RT-IMS [criterion (ii)] (**Table 1**). The correlation between baseline ICC and post-RT-IMS was 0.432 (**Figure 2**), reflecting the relative lack of overlap between the number of features that satisfied both criteria (i) and (ii). Agglomerative clustering revealed 18 groups of pairwise correlated features, indicating a maximum of 18 independent feature groups. Eight of these correlation groups included at least one feature demonstrating both a high baseline ICC and a high post-RT-IMS (the ADC-independent delta-radiomics subset), indicating a maximum of eight independent features that demonstrate change post-treatment. For non-histogram-equalized b50 images, 102/107 features demonstrated a high baseline ICC, while only three demonstrated a high post-RT-IMS. The number of features that satisfied both criteria was further reduced to two. The correlation between baseline ICC and post-RT-IMS was 0.133. Agglomerative clustering revealed 13 correlation groups; two of these correlation groups included one feature with both a high baseline ICC and a high post-RT-IMS (the b50 independent delta-radiomics subset), indicating a maximum of two independent features that demonstrate change post-treatment.

**Table 1:** Repeatability analysis.

	baseline ICC > 0.85	post-RT-IMS > 0.85	baseline ICC > 0.85, post-RT-IMS > 0.85	PCC
**ADC**	101	43	43	0.432
**ADC (histogram**-**equalized)**	103	14	14	0.288
**b50**	102	3	2	0.133
**b50 (histogram**-**equalized)**	102	12	11	0.247

Summary of the number of features that had baseline ICC >0.85 (column 1) and post-RT-IMS >0.85 (column 2) or satisfied both criteria (column 3). The correlation between the baseline ICC and post-RT-IMS (PCC) is presented for each image type in column 4.

Histogram equalization had little effect on the number of features that demonstrated high baseline ICC (103 and 102 for the ADC maps and b50 images, respectively). However, histogram equalization reduced the number of features that satisfy both criteria (i) and (ii) for the ADC images to 14 (removing nearly all second-order features except glcmMaximumProbability) but increased the number for the b50 images to 11. The correlation between baseline ICC and post-RT-IMS for histogram-equalized ADC maps and b50 images was 0.288 and 0.247, respectively (**Figure 2**). Agglomerative clustering revealed 17 and 18 correlation groups for the histogram-equalized ADC maps and b50 images, respectively, of which six and five groups included at least one feature that satisfied both criteria.

The baseline ICC, post-RT-IMS, *s*_w_bs__, *s*_b_bs__, and *s*_w_rt__ are shown for the independent delta-radiomics subset for each image contrast in **Supplementary Material D.**

**Figure 3:**
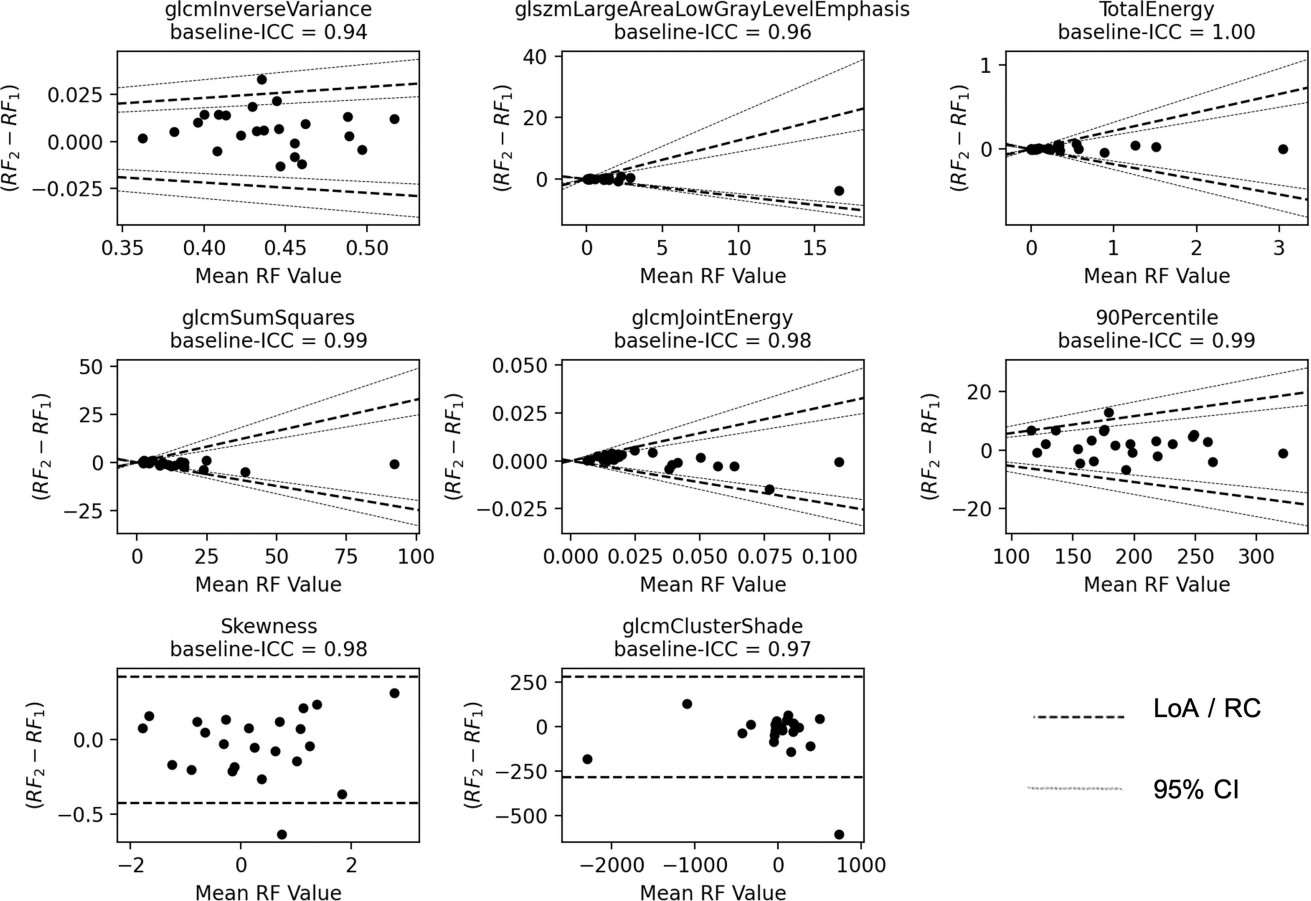
Bland–Altman plots. The vertical axis shows the difference between the two radiomic feature (RF) values and the horizontal axis shows the mean value. The LoA and their 95% confidence intervals (CI) are shown as dashed and dotted lines, respectively. For Skewness and glcmClusterShade, the repeatability coefficient (RC) is shown instead.

**Figure 4:**
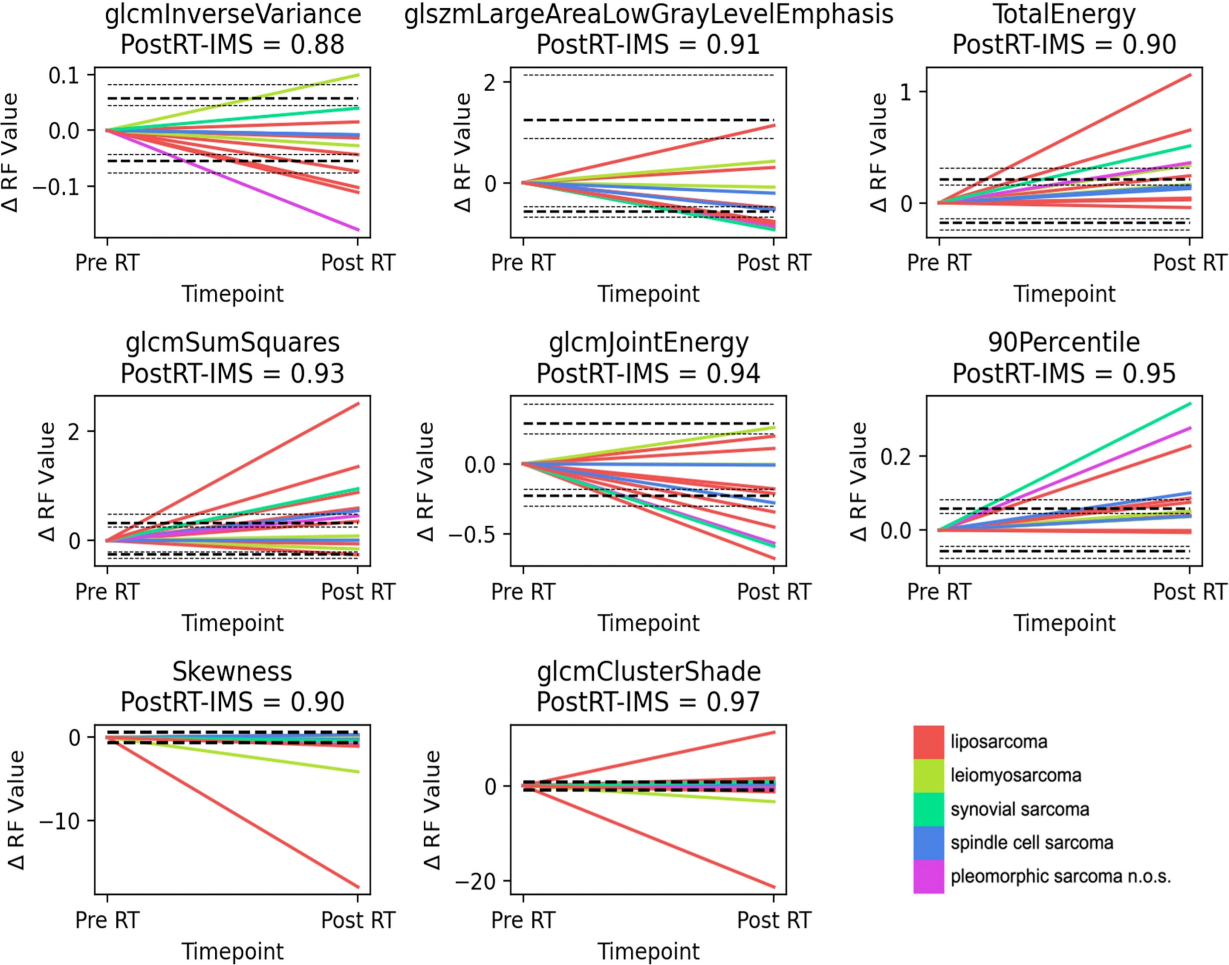
Delta-radiomics plots. Plots showing the fractional change in the radiomic feature value from the first baseline scan to post-radiotherapy treatment, for the ADC-based independent delta-radiomics subset. Interestingly, for a number of features (90 Percentile, Total Energy, and glcmSumSquares), there is a general and significant increase in these biomarkers after treatment for a number of patients, which renders them promising candidates for further exploration. The LoA and their 95% confidence intervals (CI) are shown as dashed and dotted lines, respectively. For Skewness and glcmClusterShade, the repeatability coefficient is shown instead. n.o.s., not otherwise specified.

As the non-histogram-equalized ADC maps returned the highest number of features that satisfied both criteria, these features are explored in more detail. Bland–Altman plots are shown for the ADC-independent delta-radiomics subset in **Figure 3**. The vertical axis shows the difference between the radiomic feature values across measurement 1 and measurement 2, and the horizontal axis shows the mean value between the two measurements. The plots show no clear evidence of bias and suggest that these features show good repeatability. The fractional changes in these ADC-derived radiomic features after radiotherapy are presented in **Figure 4**. Some of the radiomic features demonstrated similar treatment changes in all patients; 90percentile and TotalEnergy tended to increase after radiotherapy and glcmJointEnergy tended to decrease, while other features demonstrated a more even distribution over increase and decrease.

## Discussion

Nearly all radiomic features demonstrated good test–retest repeatability across both ADC maps and b50 images [criterion (i)]; however, the number of features that demonstrated change post-treatment was markedly lower [criterion (ii)]. Our repeatability results are in line with a previous finding by Bologna et al., where they identified 59/69 ADC-based radiomic features in STS as stable to geometrical transformations of the ROI (32). Our test–retest study uniquely assesses the stability of features in the context of a repeat baseline investigation, which includes additional clinical sources of variability such as patient positioning (33). The high repeatability found in our study may be in part due to using data from a single scanner at a single site and that sarcomas tend to be large in volume and immobile compared to many other tumor sites. Peerlings et al. found that 25%–29% of ADC-based radiomic features presented test–retest stability in other cancers across a variety of tissues, MR systems, and vendors for a standardized protocol (34).

ADC maps and histogram-equalized b50 images returned a higher number of features that demonstrate change post-treatment compared to the original low *b*-value images. MR signal is relative and can suffer from inhomogeneities which may affect radiomics analysis (33). We demonstrate that quantification *via* ADC fitting and/or histogram equalization may improve feature robustness. However, histogram equalization of the already fitted ADC values decreased the number of features that demonstrated change post-treatment. Although showing good repeatability at baseline, none of the shape features demonstrated a high post-RT-IMS, suggesting that they do not change greatly after treatment in retroperitoneal sarcomas. This is consistent with the findings of previous studies and the recommendation by the EORTC (9).

When comparing baseline repeatability alone, our analysis showed that ADC maps returned a high number of stable features [criterion (i)], which noticeably dropped when the condition of an expected significant change after treatment [criterion (ii)] was included. A similar drop in the number of stable features derived from b50 images was also observed when comparing features that satisfy both criteria, highlighting the important finding that good baseline repeatability is not necessarily indicative of sensitivity to post-treatment change. These results are further supported by the finding that the correlations between baseline ICC and post-RT-IMS in ou patient population are low (**Figure 2**). Similarly, Gudmundsson et al. demonstrated that high stability does not necessarily imply predictive power in classification models (20).

Although 107 features were calculated, only a maximum of 18 linearly independent groups were identified for the features, suggesting that many of the radiomic features are highly correlated, consistent with the results found in the literature (15, 35). For the features that demonstrate change post-treatment, only a maximum of eight linearly independent features were found.

When forming an independent delta-radiomics subset, we chose to explore the features with the highest post-RT-IMS from each correlation group. In this set, several of the features (90percentile, TotalEnergy, and glcmJointEnergy) show similar changes post-treatment (**Figure 4**), which could be showing a treatment effect. Other features demonstrate both increases and decreases for different patients, and this may be representative of the heterogeneous response typical of these tumor types or may be due to histological differences. Although many of the larger changes following treatment are shown by liposarcoma, synovial sarcoma, and pleomorphic sarcoma n.o.s., the sample size is too small to draw correlations with histology.

There are limitations to our study. Radiomic features have been shown to have varying stabilities with different image pre-processing and different settings used for feature extraction, and sensitivity to intrascanner variation (15, 33, 35–39). We kept a fixed bin-width throughout our analysis and did not use any further image pre-processing; it is possible that applying different pre-processing techniques will identify different radiomic features for treatment response evaluation that are stable and sensitive. Furthermore, our study utilized data from a single center with a single rater, and future multicenter studies could further elucidate the reproducibility of radiomic features at different imaging centers and investigate the effect of rater intraobserver variability. Apart from the in-house software used to draw ROIs, all the toolboxes used in this study are open-source and the settings used are detailed.

In conclusion, our data suggest that although nearly all DWI-based radiomic features demonstrate good baseline test-retest repeatability in STS, only a subset of features demonstrate significant change after radiotherapy. By introducing a new measure of radiomic feature stability (the post-RT-IMS), we show that good baseline repeatability does not necessarily imply a good ability to measure change post-treatment. Furthermore, we identify a range of ADC-based radiomic features that demonstrate change post-treatment, encouraging further investigation into their suitability as response markers.

## Data Availability Statement

The data from the present study are available in the ICR’s XNAT repository. Access requests will be granted depending on appropriate regulatory and institutional approvals upon contacting the corresponding author.

## Ethics Statement

The studies involving human participants were reviewed and approved by The Royal Marsden Hospital Committee for Clinical Research and approval from a national Research Ethics Committee (East of England—Cambridge East Research Ethics Committee). The patients/participants provided their written informed consent to participate in this study.

## Author Contributions

IT, MB, JW, AM, DS, KT, DC, D-MK, AA, PH, and CM: literature search. MB, JW, and IT: figures. IT, MB, JW, AM, DS, KT, DC, and CM: study design. JW, AM, DS, KT, DC, and CM: data acquisition. IT, MB, JW, DC, MO, and CM: data analysis. IT, MB, JW, MO, JO’C, AA, PH, SZ, DC, D-MK, and CM: data interpretation. IT, MB, and JW: software development. IT, MB, JW, DC, D-MK, MO, JO’C, UO, PH, SZ, and CM: article writing. All authors contributed to the article and approved the submitted version.

## Funding

We acknowledge support from the David & Ruth Lewis Family Charitable Trust, Sarcoma UK (grant number SUK105.2018). Cancer Research UK (https://www.cancerresearchuk.org/) and Engineering and Physical Sciences Research Council support to the Cancer Imaging Centre at Institute of Cancer Research and Royal Marsden Hospital in association with Medical Research Council and Department of Health C1060/A10334, C1060/A16464 and National Health Service funding to the National Institute for Health Research (https://www.nihr.ac.uk/) Biomedical Research Centre, Clinical Research Facility in Imaging and the Cancer Research Network. This report was independent research funded by the National Institute for Health Research. The views expressed in this publication are those of the author(s) and not necessarily those of the NHS, the National Institute for Health Research or the Department of Health.

## Conflict of Interest

The authors declare that the research was conducted in the absence of any commercial or financial relationships that could be construed as a potential conflict of interest.

## Publisher’s Note

All claims expressed in this article are solely those of the authors and do not necessarily represent those of their affiliated organizations, or those of the publisher, the editors and the reviewers. Any product that may be evaluated in this article, or claim that may be made by its manufacturer, is not guaranteed or endorsed by the publisher.

## References

[1] Clark MA Fisher C Judson I Meirion Thomas J. Soft-Tissue Sarcomas in Adults. New Engl J Med (2005) 353:701–11. doi: 10.1056/NEJMra041866 16107623

[2] Eisenhauer EA Therasse P Bogaerts J Schwartz LH Sargent D Ford R. New Response Evaluation Criteria in Solid Tumours: Revised RECIST Guideline (Version 1.1). Eur J Cancer (2009) 45:228–47. doi: 10.1016/j.ejca.2008.10.026 19097774

[3] Du XH Wei H Zhang P Yao WT Cai QQ. Heterogeneity of Soft Tissue Sarcomas and Its Implications in Targeted Therapy. Front Oncol (2020) 10:564852. doi: 10.3389/fonc.2020.564852 33072594 PMC7538626

[4] Stahl R Wang T Lindner LH Abdel-Rahman S Santl M Reiser MF. Comparison of Radiological and Pathohistological Response to Neoadjuvant Chemotherapy Combined With Regional Hyperthermia (RHT) and Study of Response Dependence on the Applied Thermal Parameters in Patients With Soft Tissue Sarcomas (STS). Int J Hyperther (2009) 25:289–98. doi: 10.1080/02656730902873616 19670096

[5] Canter RJ Martinez SR Tamurian RM Wilton M Li CS Ryu J. Radiographic and Histologic Response to Neoadjuvant Radiotherapy in Patients With Soft Tissue Sarcoma. Ann Surg Oncol (2010) 17:2578–84. doi: 10.1245/s10434-010-1156-3 PMC2941714 20556523

[6] Stacchiotti S Collini P Messina A Morosi C Barisella M Bertulli R. High-Grade Soft-Tissue Sarcomas: Tumor Response Assessment - Pilot Study to Assess the Correlation Between Radiologic and Pathologic Response by Using RECIST and Choi Criteria. Radiology (2009) 251:447–56. doi: 10.1148/radiol.2512081403 19261927

[7] Miki Y Ngan S Clark JCM Akiyama T Choong PFM. The Significance of Size Change of Soft Tissue Sarcoma During Preoperative Radiotherapy. Eur J Surg Oncol (2010) 36:678–83. doi: 10.1016/j.ejso.2010.05.021 20547446

[8] Roberge D Skamene T Nahal A Turcotte RE Powell T Freeman C. Radiological and Pathological Response Following Pre-Operative Radiotherapy for Soft-Tissue Sarcoma. Radiothe Oncol (2010) 97:404–7. doi: 10.1016/j.radonc.2010.10.007 21040989

[9] Messiou C Bonvalot S Gronchi A Vanel D Meyer M Robinson P. Evaluation of Response After Pre-Operative Radiotherapy in Soft Tissue Sarcomas; The European Organisation for Research and Treatment of Cancer - Soft Tissue and Bone Sarcoma Group (EORTC - STBSG) and Imaging Group Recommendations for Radiological Examina. Eur J Cancer (2016) 56:37–44. doi: 10.1016/j.ejca.2015.12.008 26802529

[10] Winfield JM Miah AB Strauss D Thway K Collins DJ DeSouza NM. Utility of Multi-Parametric Quantitative Magnetic Resonance Imaging for Characterization and Radiotherapy Response Assessment in Soft-Tissue Sarcomas and Correlation With Histopathology. Front Oncol (2019) 9:280. doi: 10.3389/fonc.2019.00280 31106141 PMC6494941

[11] Koh DM Collins DJ. Diffusion-Weighted MRI in the Body: Applications and Challenges in Oncology. Am J Roentgenol (2007) 188:1622–35. doi: 10.2214/AJR.06.1403 17515386

[12] Tsien C Cao Y Chenevert T. Clinical Applications for Diffusion Magnetic Resonance Imaging in Radiotherapy. Semin Radiat Oncol (2014) 24:218–26. doi: 10.1016/j.semradonc.2014.02.004 PMC4170230 24931097

[13] Blackledge MD Winfield JM Miah A Strauss D Thway K Morgan VA. Supervised Machine-Learning Enables Segmentation and Evaluation of Heterogeneous Post-Treatment Changes in Multi-Parametric Mri of Soft-Tissue Sarcoma. Front Oncol (2019) 9:941. doi: 10.3389/fonc.2019.00941 31649872 PMC6795696

[14] Gardin I Grégoire V Gibon D Kirisli H Pasquier D Thariat J. Radiomics: Principles and Radiotherapy Applications. Crit Rev Oncology/Hematol (2019) 138:44–50. doi: 10.1016/j.critrevonc.2019.03.015 31092384

[15] Yip SSF Aerts HJWL. Applications and Limitations of Radiomics. Phys Med Biol (2016) 61:R150–66. doi: 10.1088/0031-9155/61/13/R150 PMC4927328 27269645

[16] Corino VDA Montin E Messina A Casali PG Gronchi A Marchianò A. Radiomic Analysis of Soft Tissues Sarcomas can Distinguish Intermediate From High-Grade Lesions. J Magnet Res Imaging (2018) 47:829–40. doi: 10.1002/jmri.25791 28653477

[17] Lee SE Jung JY Nam Y Lee SY Park H Shin SH. Radiomics of Diffusion-Weighted MRI Compared to Conventional Measurement of Apparent Diffusion-Coefficient for Differentiation Between Benign and Malignant Soft Tissue Tumors. Sci Rep (2021) 11:1–10. doi: 10.1038/s41598-021-94826-w 34315971 PMC8316538

[18] Gao Y Kalbasi A Hsu W Ruan D Fu J Shao J. Treatment Effect Prediction for Sarcoma Patients Treated With Preoperative Radiotherapy Using Radiomics Features From Longitudinal Diffusion-Weighted MRIs. Phys Med Biol (2020) 65. doi: 10.1088/1361-6560/ab9e58 32554891

[19] O’Connor JPB Aboagye EO Adams JE Aerts HJWL Barrington SF Beer AJ. Imaging Biomarker Roadmap for Cancer Studies. Nat Rev Clin Oncol (2017) 14:169–86. doi: 10.1038/nrclinonc.2016.162 PMC5378302 27725679

[20] Gudmundsson S Runarsson TP Sigurdsson S. Test-Retest Reliability and Feature Selection in Physiological Time Series Classification. Comput Methods Prog Biomed (2012) 105:50–60. doi: 10.1016/j.cmpb.2010.08.005 20864206

[21] Harris CR Millman KJ van der Walt SJ Gommers R Virtanen P Cournapeau D. Array Programming With NumPy. Nature (2020) 585:357–62. doi: 10.1038/s41586-020-2649-2 PMC7759461 32939066

[22] Lowekamp BC Chen DT Ibáñez L Blezek D. The Design of simpleITK. Front Neuroinformat (2013) 7:45. doi: 10.3389/fninf.2013.00045 PMC3874546 24416015

[23] Yaniv Z Lowekamp BC Johnson HJ Beare R. SimpleITK Image-Analysis Notebooks: A Collaborative Environment for Education and Reproducible Research. J Dig Imaging (2018) 31:290–303. doi: 10.1007/s10278-017-0037-8 PMC5959828 29181613

[24] Thrussell I Winfield J Orton M Miah A Zaidi S Arthur A. Investigating the Correlation and Repeatability of Radiomic Features Derived From Apparent Diffusion Coefficient Maps of Soft-Tissue Sarcoma. Proc Int Soc Mag Res Med (2020), 4817.

[25] van Griethuysen JJM Fedorov A Parmar C Hosny A Aucoin N Narayan V. Computational Radiomics System to Decode the Radiographic Phenotype. Cancer Res (2017) 77:e104–7. doi: 10.1158/0008-5472.CAN-17-0339 PMC5672828 29092951

[26] Euser AM Dekker FW le Cessie S. A Practical Approach to Bland-Altman Plots and Variation Coefficients for Log Transformed Variables. J Clin Epidemiol (2008) 61:978–82. doi: 10.1016/j.jclinepi.2007.11.003 18468854

[27] Bland JM Altman DG. Statistical Methods for Assessing Agreement Between Two Methods of Clinical Measurement. Int J Nurs Stud (2010) 47:931–6. doi: 10.1016/j.ijnurstu.2009.10.001 2868172

[28] van Timmeren JE Leijenaar RTH van Elmpt W Wang J Zhang Z Dekker A. Test–Retest Data for Radiomics Feature Stability Analysis: Generalizable or Study-Specific? Tomography (2016) 2:361–5. doi: 10.18383/j.tom.2016.00208 PMC6037932 30042967

[29] Hunter JD. Matplotlib: A 2d Graphics Environment. Comp Sci Eng (2007) 9:90–5. doi: 10.1109/MCSE.2007.55

[30] Waskom M. Seaborn: Statistical Data Visualization. J Open Source Soft (2021) 6:3021. doi: 10.21105/joss.03021

[31] Virtanen P Gommers R Oliphant TE Haberland M Reddy T Cournapeau D. SciPy 1.0: Fundamental Algorithms for Scientific Computing in Python. Nat Methods (2020) 17:261–72. doi: 10.1038/s41592-019-0686-2 PMC7056644 32015543

[32] Bologna M Corino VDA Montin E Messina A Calareso G Greco FG. Assessment of Stability and Discrimination Capacity of Radiomic Features on Apparent Diffusion Coefficient Images. J Dig Imaging (2018) 31:879–94. doi: 10.1007/s10278-018-0092-9 PMC6261192 29725965

[33] Schwier M van Griethuysen J Vangel MG Pieper S Peled S Tempany C. Repeatability of Multiparametric Prostate MRI Radiomics Features. Sci Rep (2019) 9:1–16. doi: 10.1038/s41598-019-45766-z 31263116 PMC6602944

[34] Peerlings J Woodruff HC Winfield JM Ibrahim A van Beers BE Heerschap A. Stability of Radiomics Features in Apparent Diffusion Coefficient Maps From a Multi-Centre Test-Retest Trial. Sci Rep (2019) 9:1–10. doi: 10.1038/s41598-019-41344-5 30886309 PMC6423042

[35] Traverso A Wee L Dekker A Gillies R. Repeatability and Reproducibility of Radiomic Features: A Systematic Review. Int J Radiat OncologyBiologyPhys (2018) 102:1143–58. doi: 10.1016/j.ijrobp.2018.05.053 PMC6690209 30170872

[36] Traverso A Kazmierski M Shi Z Kalendralis P Welch M Nissen HD. Stability of Radiomic Features of Apparent Diffusion Coefficient (ADC) Maps for Locally Advanced Rectal Cancer in Response to Image Pre-Processing. Phys Med (2019) 61:44–51. doi: 10.1016/j.ejmp.2019.04.009 31151578

[37] Cattell R Chen S Huang C. Robustness of Radiomic Features in Magnetic Resonance Imaging: Review and a Phantom Study. Visual Comp Indust Biomed Art (2019) 2:19. doi: 10.1186/s42492-019-0025-6 PMC7099536 32240418

[38] Ford J Dogan N Young L Yang F. Quantitative Radiomics: Impact of Pulse Sequence Parameter Selection on MRI-Based Textural Features of the Brain. Contrast Media Mol Imaging (2018) 2018:9. doi: 10.1155/2018/1729071 PMC6091359 30154684

[39] Yang F Dogan N Stoyanova R Ford JC. Evaluation of Radiomic Texture Feature Error Due to MRI Acquisition and Reconstruction: A Simulation Study Utilizing Ground Truth. Phys Med (2018) 50:26–36. doi: 10.1016/j.ejmp.2018.05.017 29891091

