# Supplementary material for: Radiomic Features From Diffusion-Weighted MRI of Retroperitoneal Soft-Tissue Sarcomas Are Repeatable and Exhibit Change After Radiotherapy
Source: Front Oncol. 2022 Jul 18;12:899180. doi: 10.3389/fonc.2022.899180 (PMC9343063; doi:10.3389/fonc.2022.899180)
Supplement: Supplementary file 1 [file Table_1.docx]

**Supplementary Material A**

Summary of patients, histological sub-types, and acquisition of repeated baseline and/or post-radiotherapy MRI examinations. Y = YES, F = FEMALE, M = MALE.

| Patient | Second Baseline | Post Treatment Scan | Histological subtype | Age | Sex |
| --- | --- | --- | --- | --- | --- |
| a | Y | NO | liposarcoma | 67 | F |
| b | Y | NO | liposarcoma | 68 | F |
| c | Y | NO | liposarcoma | 74 | F |
| d | Y | Y | leiomyosarcoma | 59 | M |
| e | Y | NO | liposarcoma | 68 | M |
| f | Y | NO | liposarcoma | 57 | M |
| g | Y | Y | leiomyosarcoma | 62 | F |
| h | Y | Y | liposarcoma | 65 | M |
| i | Y | Y | liposarcoma | 61 | F |
| j | Y | NO | liposarcoma | 51 | M |
| k | Y | Y | liposarcoma | 66 | M |
| l | Y | Y | liposarcoma | 51 | F |
| m | Y | NO | liposarcoma | 58 | M |
| n | Y | NO | liposarcoma | 67 | F |
| o | Y | NO | liposarcoma | 58 | M |
| p | Y | NO | liposarcoma | 72 | M |
| q | Y | Y | liposarcoma | 51 | M |
| r | Y | Y | leiomyosarcoma | 76 | M |
| s | Y | Y | spindle cell sarcoma | 64 | F |
| t | Y | Y | spindle cell sarcoma | 42 | F |
| u | Y | Y | pleomorphic sarcoma,  not otherwise specified | 64 | F |
| v | Y | Y | synovial sarcoma | 59 | F |
| w | NO | Y | leiomyosarcoma | 72 | M |
| x | Y | Y | liposarcoma | 68 | F |
