# Supplementary figures and images for: Radiomic Features From Diffusion-Weighted MRI of Retroperitoneal Soft-Tissue Sarcomas Are Repeatable and Exhibit Change After Radiotherapy
Source: Front Oncol. 2022 Jul 18;12:899180. doi: 10.3389/fonc.2022.899180 (PMC9343063; doi:10.3389/fonc.2022.899180)

**Supplementary Material B**

Visual representation of the baseline-ICC and the postRT-IMS.

**
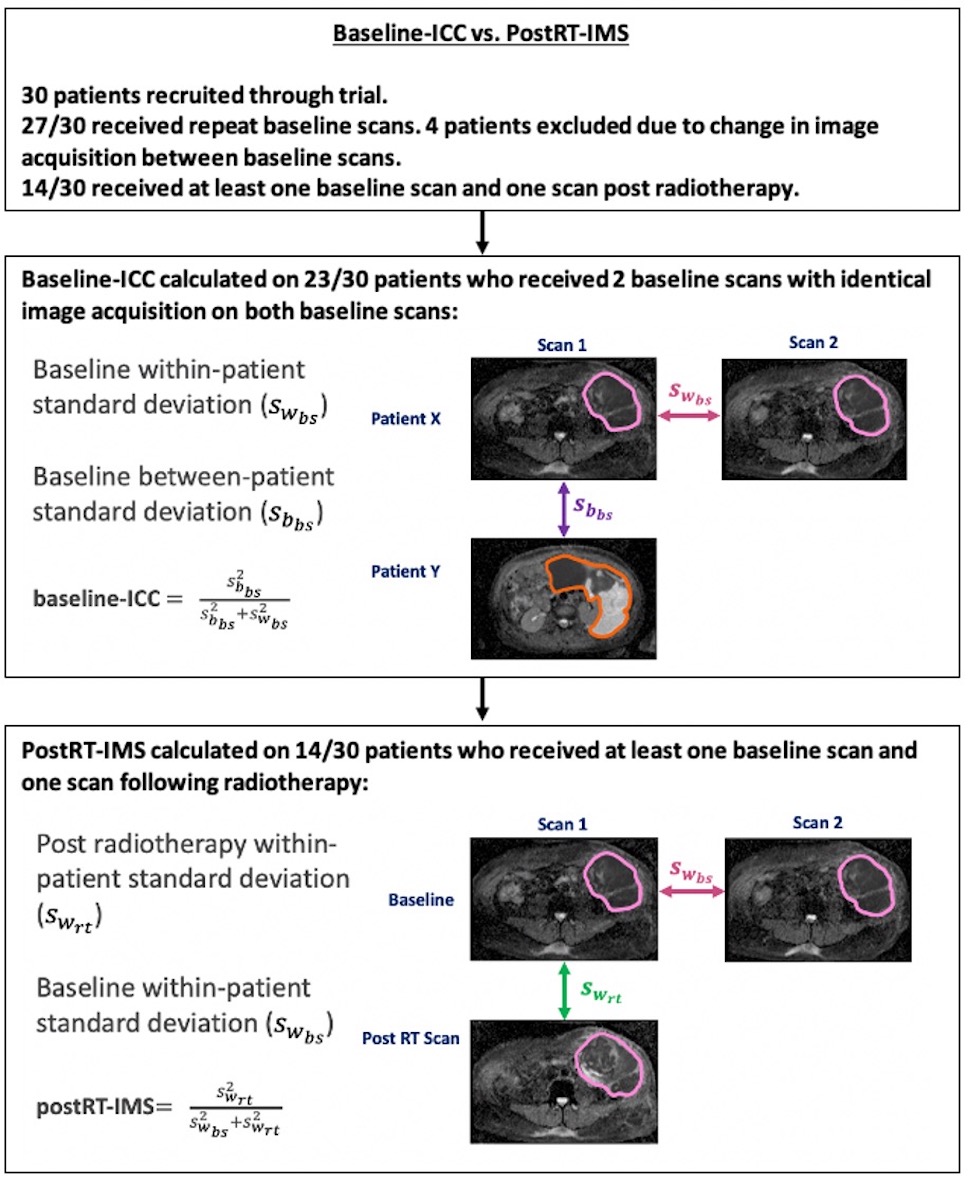
**

Supplement: Supplementary file 2 [file Table_2.docx]
