# Supplementary material for: Radiomic Features From Diffusion-Weighted MRI of Retroperitoneal Soft-Tissue Sarcomas Are Repeatable and Exhibit Change After Radiotherapy
Source: Front Oncol. 2022 Jul 18;12:899180. doi: 10.3389/fonc.2022.899180 (PMC9343063; doi:10.3389/fonc.2022.899180)
Supplement: Supplementary file 3 [file Table_3.docx]

**Supplementary Material C**

Correlation Groups for non-histogram-equalised ADC-based radiomic features.

1^st^ column = Cluster Group

2^nd^ column = Features with baseline-ICC <= 0.85

3^rd^ column = Features with baseline-ICC > 0.85 and postRT-IMS <= 0.85

4^th^ column = Features with baseline-ICC > 0.85 and postRT-IMS > 0.85

**Bold features** are in the ADC independent delta-radiomics subset

| 1 | ﻿glszmSizeZoneNonUniformity-Normalized,  ﻿glszmSmallAreaEmphasis | ﻿gldmDependenceNon-UniformityNormalized,  gldmDependenceVariance,  gldmSmallDependence-Emphasis, | ﻿﻿glcmContrast,  glcmDifferenceAverage,  glcmDifferenceEntropy,  glcmDifferenceVariance,  glcmId,  glcmIdm,  **glcmInverseVariance**,  gldmLargeDependence-Emphasis,  glrlmLongRunEmphasis,    glrlmRunLengthNon-UniformityNormalized,  glrlmRunPercentage,    glrlmRunVariance,  glrlmShortRunEmphasis,  glszmLargeAreaEmphasis,  glszmZonePercentage,  glszmZoneVariance |
| --- | --- | --- | --- |
| 2 |  | glszmLargeAreaHigh-GrayLevelEmphasis |  |
| 3 |  | shapeFlatness |  |
| 4 |  | shapeSphericity |  |
| 5 |  | 10Percentile |  |
| 6 |  | shapeElongation |  |
| 7 | ﻿glcmIdmn | glcmIdn,  ngtdmCoarseness,  shapeSurfaceVolumeRatio |  |
| 8 | ﻿Minimum | ﻿gldmLargeDependence-LowGrayLevelEmphasis,  gldmLowGrayLevelEmphasis,  gldmSmallDependence-LowGrayLevelEmphasis,  glrlmLongRunLowGray-LevelEmphasis,  glrlmLowGrayLevel-RunEmphasis,    glrlmShortRunLowGray-LevelEmphasis,  glszmLowGrayLevel-ZoneEmphasis,    glszmSmallAreaLowGray-LevelEmphasis | **﻿glszmLargeAreaLow-GrayLevelEmphasis** |
| 9 | ﻿Maximum | Range,  ngtdmComplexity |  |
| 10 |  | ﻿gldmDependenceNon-Uniformity,  glrlmGrayLevelNonUniformity,  glrlmRunLengthNonUniformity,  glszmGrayLevelNonUniformity,  glszmSizeZoneNonUniformity,  shapeLeastAxisLength,    shapeMajorAxisLength,  shapeMaximum2D-DiameterColumn,  shapeMaximum2D-DiameterRow,    shapeMaximum2D-DiameterSlice,    shapeMaximum3DDiameter,  shapeMeshVolume,  shapeMinorAxisLength,  shapeSurfaceArea,  shapeVoxelVolume | ﻿Energy,  **TotalEnergy,**  gldmGrayLevelNon-Uniformity |
| 11 |  | ngtdmBusyness |  |
| 12 |  | ngtdmStrength |  |
| 13 |  | ﻿ngtdmContrast | ﻿InterquartileRange,  MeanAbsoluteDeviation,    RobustMeanAbsolute-Deviation,  Variance,  glcmClusterProminence,  glcmClusterTendency,  **glcmSumSquares,**  gldmGrayLevelVariance,  glrlmGrayLevelVariance,  glszmGrayLevelVariance |
| 14 | ﻿glcmMCC | glcmCorrelation,    glcmImc1,  glcmImc2,  gldmDependenceEntropy,  glrlmRunEntropy,  glszmZoneEntropy |  |
| 15 |  | ﻿Kurtosis,    glszmGrayLevelNon-UniformityNormalized | ﻿Entropy,  Uniformity,  **glcmJointEnergy,**  glcmJointEntropy,  glcmMaximumProbability,  glcmSumEntropy,    glrlmGrayLevelNon-UniformityNormalized |
| 16 |  | ﻿glcmAutocorrelation,  glcmJointAverage,  glcmSumAverage,  gldmHighGrayLevelEmphasis,    gldmLargeDependence-HighGrayLevelEmphasis,    gldmSmallDependence-HighGrayLevelEmphasis,    glrlmHighGrayLevel-RunEmphasis,    glrlmLongRunHighGray-LevelEmphasis,    glrlmShortRunHighGray-LevelEmphasis,  glszmHighGrayLevel-ZoneEmphasis,    glszmSmallAreaHighGray-LevelEmphasis | ﻿**90Percentile**,  Mean,  Median,  RootMeanSquared |
| 17 |  |  | **﻿glcmClusterShade** |
| 18 |  |  | **﻿Skewness** |

﻿
