# Supplementary material for: Radiomic Features From Diffusion-Weighted MRI of Retroperitoneal Soft-Tissue Sarcomas Are Repeatable and Exhibit Change After Radiotherapy
Source: Front Oncol. 2022 Jul 18;12:899180. doi: 10.3389/fonc.2022.899180 (PMC9343063; doi:10.3389/fonc.2022.899180)
Supplement: Supplementary file 4 [file Table_4.docx]

**Supplementary Material D**

Summary statistics for the independent delta-radiomics subset for each image type, including the baseline-ICC, postRT-IMS, baseline within-patient variance ($s_{w_{bs}}$), baseline between-patient variance ($s_{b_{bs}}$), post RT within-patient variance ($s_{w_{rt}}$)

|  |  | **baseline-ICC** | **postRT-IMS** | $s_{w_{bs}}$ | $s_{b_{bs}}$ | $s_{w_{rt}}$ |
| --- | --- | --- | --- | --- | --- | --- |
| **ADC** | glcmInverseVariance | 0.944 | 0.885 | 0.020 | 0.083 | 0.056 |
|  | glszmLargeAreaLowGrayLevelEmphasis | 0.960 | 0.910 | 0.294 | 1.436 | 0.934 |
|  | TotalEnergy | 0.999 | 0.903 | 0.071 | 2.029 | 0.217 |
|  | glcmSumSquares | 0.988 | 0.932 | 0.102 | 0.936 | 0.377 |
|  | glcmJointEnergy | 0.985 | 0.938 | 0.092 | 0.735 | 0.357 |
|  | 90Percentile | 0.994 | 0.950 | 0.020 | 0.260 | 0.089 |
|  | Skewness | 0.981 | 0.905 | 0.153 | 1.096 | 0.367 |
|  | glcmClusterShade | 0.970 | 0.968 | 101.629 | 579.121 | 158.368 |
| **ADC** | Variance | 0.986 | 0.926 | 0.108 | 0.908 | 0.380 |
| **(histogram equalised)** | Uniformity | 0.988 | 0.934 | 0.051 | 0.456 | 0.191 |
|  | glcmMaximumProbability | 0.918 | 0.855 | 0.076 | 0.255 | 0.184 |
|  | Energy | 0.999 | 0.903 | 0.0712 | 2.03 | 0.217 |
|  | 90Percentile | 0.994 | 0.950 | 0.020 | 0.260 | 0.089 |
|  | Skewness | 0.981 | 0.905 | 0.153 | 1.096 | 0.367 |
| **b50** | glcmCorrelation | 0.975 | 0.947 | 0.021 | 0.132 | 0.089 |
|  | glcmClusterShade | 0.959 | 0.945 | 3596.277 | 17322.735 | 11142.589 |
| **b50** | glcmMCC | 0.957 | 0.917 | 0.016 | 0.076 | 0.054 |
| **(histogram equalised)** | gldmLargeDependenceLowGrayLevelEmphasis | 0.933 | 0.876 | 0.134 | 0.502 | 0.358 |
|  | glrlmGrayLevelVariance | 0.977 | 0.875 | 0.006 | 0.039 | 0.016 |
|  | glcmClusterShade | 0.868 | 0.873 | 48.090 | 123.485 | 118.967 |
